# A commentary on ‘Identification of the consistently differential expressed hub mRNAs and proteins in lung adenocarcinoma and construction of the prognostic signature: a multidimensional analysis’

**DOI:** 10.1097/JS9.0000000000001425

**Published:** 2024-04-04

**Authors:** Jospin Sindya, Elumalai Perumal, Krishnamoorthy Gunasekaran

**Affiliations:** a Cancer Genomics Laboratory, Centre for Global Health Research, Saveetha Medical College, Saveetha Institute of Medical and Technical Sciences, Saveetha University, Chennai, India; b Department of Medical Biochemistry, College of Health Sciences, Dambi Dollo University, Kelam Welega Zone, Oromia Region, Ethiopia

*Dear Editor,*

The innovative approach, thorough analysis, reliable predictions, validations, and outcomes of a recent study on ‘Identification of consistently differential expressed hub mRNAs and proteins in lung adenocarcinoma and construction of the prognostic signature: a multidimensional analysis’ by Liu *et al*.^1^ has captured our interest immensely. The study emphasized differentially expressed eight hub messenger RNAs (mRNAs) and validated their universal acceptability. The novelty of the study lies in the identification of GPI (glucose-6-phosphate isomerase), a plasma protein, as a diagnostic and prognostic marker in lung adenocarcinoma (LUAD).

Genomic data for the study was obtained from The Cancer Genome Atlas (TCGA) database and Gene Expression Omnibus (GEO) database for Western and Chinese populations, respectively. For the Western population, proteomics data was retrieved from the Clinical Proteomic Tumor Analysis Consortium (CPTAC) data portal. For the Chinese population, iProx Consortium was used to derive proteomics data. These reliable sources of databases build a strong base for the study. By including analysis of differential expression of mRNAs and proteins across genders and races, the dynamic and versatile nature of the study was held high. Differentially expressed eight hub mRNAs were identified to be prognosis-related and were further validated. A survival model was built using the hub mRNAs. The author broadened the model by incorporating Cancer stage and smoking status into the survival model in order to acknowledge the significance of clinical and pathological data in cancer prognosis. Since clinical trials targeting EGFR (epidermal growth factor receptor) mutation have been shown to improve the quality of life in lung cancer patients^2^, EGFR mutation data was also included in constructing the survival model. The survival model thus constructed stands robust and consistent in its prediction accuracy.

Pathway enrichment analysis revealed involvement in extracellular exosomes and membranes in GO (Gene Ontology) analysis, and glutathione metabolism was found to be most significant in KEGG (Kyoto Encyclopedia of Genes and Genomes) analysis. Among the eight hub mRNAs/proteins, GPI, a key enzyme in glycolysis, was found to be significantly upregulated in the plasma and tissue of patients with lung cancer. Over the past few years, GPI was found to act as a biomarker in some cancer types including Breast cancer^3^. GPI expression in various cancer types is depicted in Figure 1. The potential mechanism of GPI in LUAD was analyzed by performing Correlation analysis. High plasma GPI expression was positively correlated with poor progression, and elevated GPI expression in tumor tissues was associated with vascular anomalies.

**Figure 1:**
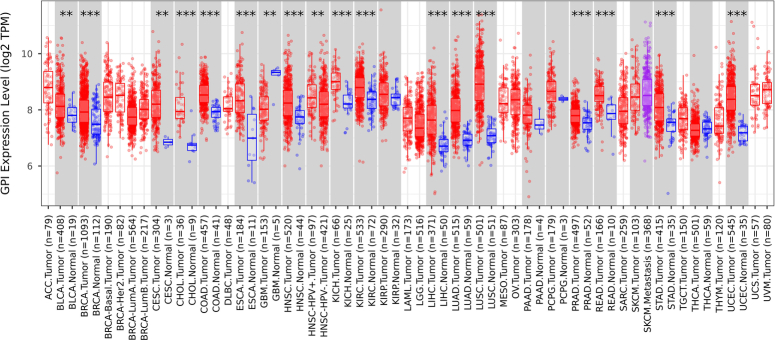
Analysis of GPI (glucose-6-phosphate isomerase) expression in different cancers in TIMER2.0 database (http://timer.cistrome.org/)^4^. In TIMER2.0 database analysis, GPI is highly expressed in Breast Cancer (BRCA), Cervical Squamous Cell Carcinoma and Endocervical Adenocarcinoma (CESC), Cholangiocarcinoma (CHOL), Colon Adenocarcinoma (COAD), Esophageal carcinoma (ESCA), Head and Neck Squamous cell carcinoma (HNSC), Kidney Chromophobe (KICH), Kidney renal cell carcinoma (KIRC), Liver hepatocellular carcinoma (LIHC), Lung adenocarcinoma (LUAD), Lung squamous cell carcinoma (LUSC), Prostate adenocarcinoma (PRAD), Rectum adenocarcinoma (READ), Stomach adenocarcinoma (STAD), and Uterine corpus endometrial carcinoma (UCEC). The gray columns display the upregulated or downregulated gene expression in tumors compared to normal tissues. Stars denote statistical significance by the Wilcoxon test (*P<0.05; **P<0.01; ***P<0.001).

Biomarkers for lung cancer prediction and prognosis include PD-L1 (programmed death-ligand 1) expression, tumor mutation burden (TMB), tumor-infiltrating lymphocytes, LIPI (Lung Immune Prognostic Index), microsatellite instability, etc., though commonly used, lack unified detection standards^5^. Thus, efficient predictive and prognostic biomarkers for Lung cancer are desperately needed. Though greatly applicable, the study has its limitations such as proteomic and transcriptomic data being from different samples and the risk of bias due to different experimental methods in different studies, which are duly recognized by the author. The study with its efficient design and thorough analysis and validation, provides valuable information for future scopes in the diagnosis of LUAD and the use of GPI as a liquid biopsy biomarker, thereby enhancing feasible yet effective diagnosis and prognosis of lung cancer.

## Ethical approval

Not applicable.

## Consent

Not applicable.

## Sources of funding

Not applicable.

## Author contribution

J.S.: conceptualization, writing–original draft, writing–review and editing, and visualization; E.P.: conceptualization, writing–original draft, writing–review and editing, and supervision; K.G.: conceptualization, writing– review and editing, and supervision.

## Conflicts of interest disclosure

There are no conflicts of interest.

## Research registration unique identifying number (UIN)

Not applicable.

## Guarantor

Krishnamoorthy Gunasekaran.

## Data availability statement

Not applicable.
